# Tardy posterior interosseous nerve palsy resulting from residual dislocation of the radial head in a Monteggia fracture: a case report

**DOI:** 10.1186/1752-1947-3-9300

**Published:** 2009-11-27

**Authors:** Chul-Hyun Cho, Kyung-Jae Lee, Byung-Woo Min

**Affiliations:** 1 Department of Orthopaedic Surgery, College of Medicine, Keimyung University, Daegu, Korea

## Abstract

**Introduction:**

We report an extremely rare case of tardy posterior interosseous nerve palsy that developed 40 years after unreduced anterior dislocation of the radial head in a Monteggia fracture.

**Case presentation:**

A 46-year-old Asian woman was diagnosed with tardy posterior interosseous nerve palsy resulting from residual dislocation of the radial head in a Monteggia fracture. The patient remembered that she had sustained a fracture to the right elbow when she was 6 years old but could not remember the details of either the injury or its treatment. Intra-operatively, the posterior interosseous nerve was compressed at the radial head, wrapped around the medial side of the radial neck, and ran into the distorted supinator muscle, and was stretched. We therefore excised the radial head and performed neurolysis. The function of the right hand was normal at a follow-up examination 8 months after surgery.

**Conclusion:**

We theorize that excessive repeated motion with loss of elasticity of surrounding tissues because of long-term dislocation of the radial head may cause delayed posterior interosseous nerve palsy. It is necessary to make an accurate diagnosis and render proper treatment when a Monteggia fracture occurs, making sure that the radial head does not remain dislocated, to avoid possible posterior interosseous nerve palsy due to excessive pronation and supination even several decades later.

## Introduction

Acute posterior interosseous nerve (PIN) palsy after a Monteggia fracture has been reported fairly frequently [1-3]. However, tardy PIN palsy resulting from residual dislocation of the radial head in these fractures is extremely rare. Lichter and Jacobsen [4] first reported a case of tardy PIN palsy with a Monteggia fracture. Since then, four cases have been reported in the English literature [5-7]. We report a case of tardy PIN palsy that developed 40 years after an unreduced anterior dislocation of the radial head in a Monteggia fracture.

## Case presentation

A 46-year-old Asian woman was referred to our hospital with a history of weakness in her right hand of approximately one month's duration. She was a housewife and her symptoms developed after undertaking strenuous work helping her family prepare for a wedding. The patient remembered that she had sustained a fracture to the right elbow when she was 6 years old but could not remember the details of either the injury or its treatment.

**Figure 1:**
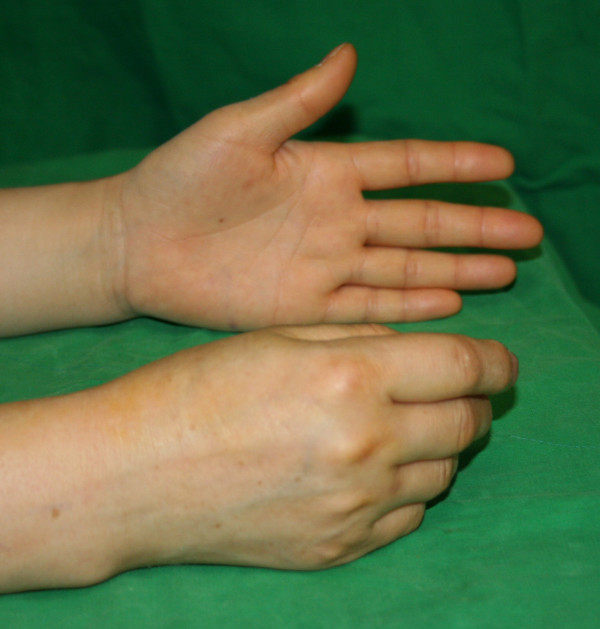
**Pre-operative clinical photo showing that the power of extension of the thumb and four fingers of her right hand was reduced**.

On physical examination, the power of extension of the thumb and four fingers of her right hand was reduced to a trace, but the power of dorsiflexion of her wrist was normal (Figure 1). Pre-operative electrodiagnostic data were compatible with PIN palsy. There was no sensory disturbance in the area supplied by the superficial radial nerve. The range of motion of her right elbow was limited, being 5° of hyperextension to 110° of flexion, 80° of pronation, and 40° of supination. A bony prominence was palpated on the anterolateral aspect of her elbow.

**Figure 2:**
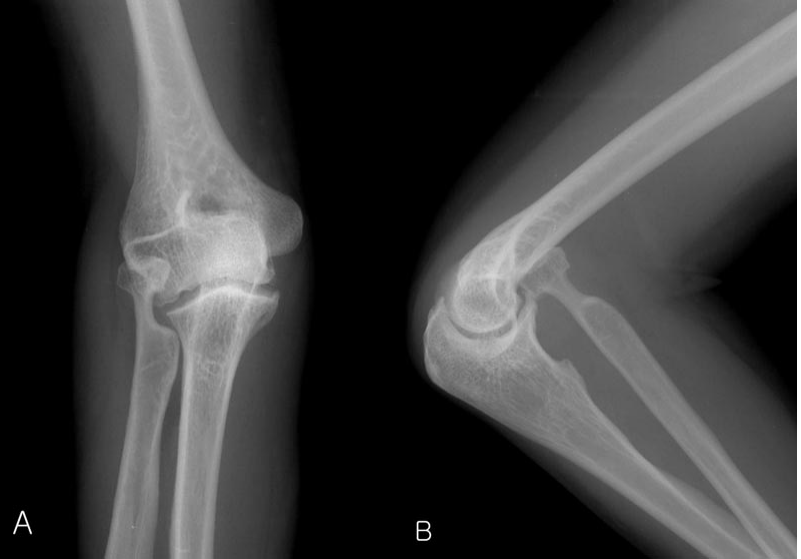
**Plain radiographs showing anterior dislocation of the radial head and elongated radial neck: (A) anteroposterior view; (B) lateral view**.

**Figure 3:**
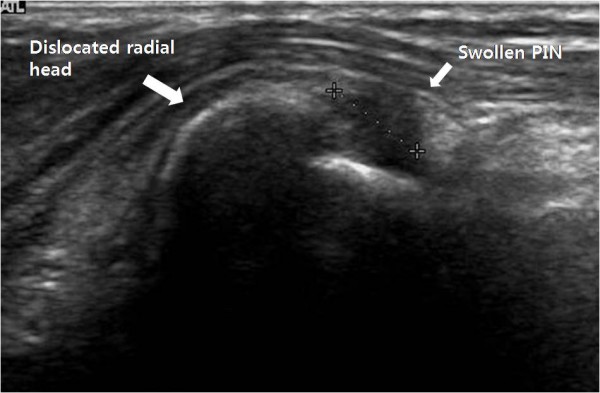
**Ultrasound image showing compression of the posterior interosseous nerve by the dislocated radial head, with focal swelling**.

Plain radiographs of her right elbow revealed an anterior dislocation of the radial head and elongated radial neck (Figure 2). Ultrasonography revealed that the posterior interosseous nerve was compressed by the dislocated radial head, and there was focal swelling from just distal to the radial head to just before it entered the supinator muscle (Figure 3).

**Figure 4:**
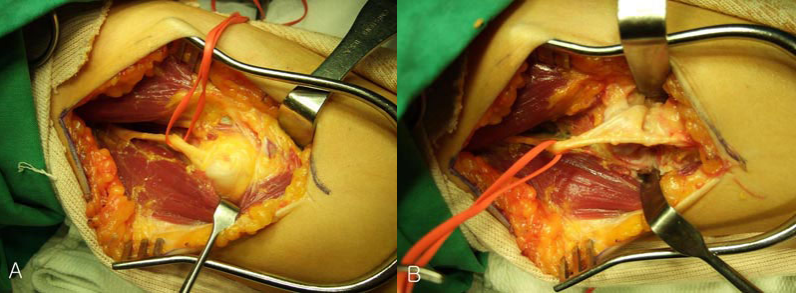
**Intra-operative findings**. (A) The radial nerve is encased in fibrotic tissue that also enveloped the posterior interosseous nerve and lay anterior and superior to the dislocated radial head. (B) After dissection, the posterior interosseous nerve is compressed at the radial head and wraps around the medial side of the radial neck.

We performed an exploration with an anterolateral curved incision of the right elbow. The radial nerve was encased in fibrotic tissue that also enveloped the PIN and lay anterior and superior to the dislocated radial head. We dissected out the nerve and fibrotic tissue. We found that the PIN was compressed at the radial head, wrapped around the medial side of the radial neck, and ran into the distorted supinator muscle (Figure 4). We found intra-operatively that the PIN was stretched. Therefore, neurolysis alone or reduction of the radial head could not release the PIN, so we excised the radial head.

The patient began to recover extension of her four fingers one month after surgery and extension of her thumb four months after surgery. The function of her right hand and electrodiagnostic data were normal at a follow-up examination 8 months after surgery.

## Discussion

Conditions that may cause nontraumatic PIN palsy include compression by the fibrous edge of the entrance or exit of the supinator, benign tumors (including lipoma, ganglion and fibroma), rheumatoid arthritis, neuralgic amyotrophy, nerve constriction, delayed paralysis as a result of residual dislocation of the radial head in a Monteggia fracture, and chronic minor repetitive motion at work [8].

Only five cases of tardy PIN palsy associated with an old unreduced radial head in Monteggia fractures have been reported in the English literature [4-7]. It is interesting that the interval between the original injury and the onset of palsy was >30 years in all cases, with a mean interval of 42.6 years (range 30-65 years). The mean time to complete recovery in those cases was 5.5 months (range 1.5-11 months) after surgical treatment of neurolysis only or resection of the radial head and neurolysis (Table 1). The interval between the injury and the onset of palsy in our patient and her time to recovery was similar to those reported in earlier cases.

**Table 1:** Cases of tardy posterior interosseous nerve palsy resulting from residual dislocation of radial head in a Monteggia fracture

Author(s)	Age (years)	Age (years) at the original injury	Interval (years)	Duration of symptoms (months)	Treatment	Length of postoperative recovery (months)
Lichter and Jacobsen, 1975 [4]	46	7	39	12	Excision of the radial head and neurolysis	2.5
Austin, 1976 [5]	72	7	65	1	Excision of the radial head and neurolysis	9
Holst-Nielsen and Jensen, 1984 [6]	46	7	39	9	Neurolysis	4
	34	4	30	24	Neurolysis	11
Hashizume *et al*., 1995 [7]	44	5	39	2.5	Excision of the radial head and neurolysis	1.5

## Conclusion

We theorize that excessive repeated motion with loss of elasticity of surrounding tissues because of dislocation of the radial head for a long period may eventually cause delayed PIN palsy.

It is necessary to make an accurate diagnosis and render proper treatment when a Monteggia fracture occurs, making sure that the radial head does not remain dislocated, to avoid PIN palsy due to excessive pronation and supination even several decades later.

## Consent

Written informed consent was obtained from the patient for publication of this case report and any accompanying images. A copy of the written consent is available for review by the Editor-in-Chief of this journal.

## Competing interests

The authors declare that they have no competing interests.

## Authors' contributions

BWM reviewed and interpreted the patient data and X-ray. CHC and KJL performed the operation, and were major contributors in writing the manuscript. All authors read and approved the final manuscript.
